# Presynaptic Inhibition of Pain and Touch in the Spinal Cord: From Receptors to Circuits

**DOI:** 10.3390/ijms22010414

**Published:** 2021-01-02

**Authors:** Antonella Comitato, Rita Bardoni

**Affiliations:** Department of Biomedical, Metabolic and Neural Sciences, University of Modena and Reggio Emilia, 41125 Modena, Italy; comitatoantonella@gmail.com

**Keywords:** sensory transmission, dorsal horn, presynaptic inhibition, GABA

## Abstract

Sensory primary afferent fibers, conveying touch, pain, itch, and proprioception, synapse onto spinal cord dorsal horn neurons. Primary afferent central terminals express a wide variety of receptors that modulate glutamate and peptide release. Regulation of the amount and timing of neurotransmitter release critically affects the integration of postsynaptic responses and the coding of sensory information. The role of GABA (γ-aminobutyric acid) receptors expressed on afferent central terminals is particularly important in sensory processing, both in physiological conditions and in sensitized states induced by chronic pain. During the last decade, techniques of opto- and chemogenetic stimulation and neuronal selective labeling have provided interesting insights on this topic. This review focused on the recent advances about the modulatory effects of presynaptic GABAergic receptors in spinal cord dorsal horn and the neural circuits involved in these mechanisms.

## 1. Introduction

**Figure 1:**
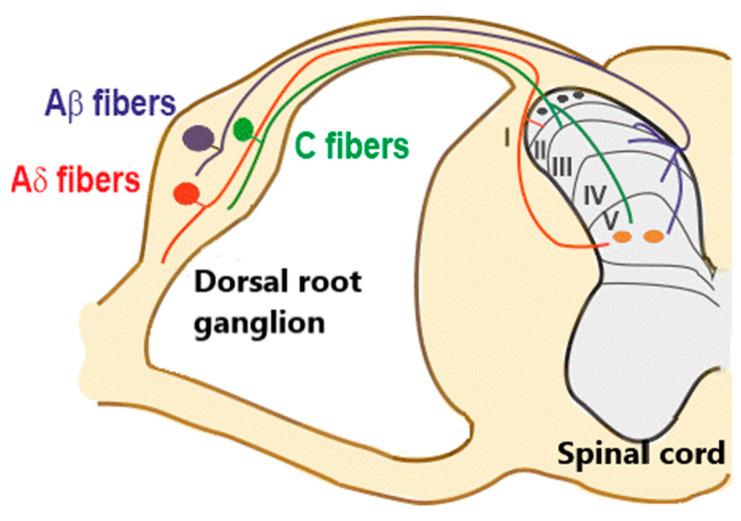
Organization of primary afferent fibers projecting to spinal cord dorsal horn. Nociceptive fibers terminate mainly in superficial dorsal horn (laminae I–II), while tactile afferents (Aβ and LT (low threshold) Aδ) project mainly to deep dorsal horn (laminae III–V). Nociceptive projection neurons are located in lamina I (black circles), while wide dynamic range neurons (orange circles), activated by both tactile and nociceptive stimuli, are localized in lamina V. Muscle afferent fibers (Aα) are not considered in this diagram.

The spinal cord dorsal horn represents the principal station where primary afferent fibers (PAFs) terminate, carrying information about several somatic sensory modalities. Primary afferent fibers transmitting pain, touch, itch, and proprioceptive inputs project to different dorsal horn laminae (laminae I–VI) (Figure 1), synapsing onto excitatory and inhibitory interneurons and projection neurons. PAFs belong to different categories (Aα, Aβ, Aδ, and C) that convey distinct sensory modalities. In particular, the fiber groups relevant to this review include: (1) myelinated Aβ and low threshold (LT) Aδ fibers, mediating innocuous mechanical sensitivity, and mainly projecting to lamina II inner, III, and IV; (2) high threshold (HT) myelinated Aδ and unmyelinated C fibers, mediating pain, thermal sensations, and itch, and mainly terminating in lamina I and II. Some nociceptive and tactile inputs also reach lamina V, where they converge on wide dynamic range neurons. Muscle afferent fibers were not considered in this review.

The variety of neurons in the spinal cord makes the investigation of dorsal horn circuits difficult. Based on the expression of distinct neurochemical markers, several classes of neurons have been identified in rodent lamina I, II, and III of the dorsal horn, exhibiting different morphologies and functional properties [1,2,3,4]. The transgenic expression of fluorescent proteins, such as EGFP or tdTomato, driven by a cell-type-specific promoter, has allowed the characterization of morphological and electrophysiological properties of specific neuron populations. Glutamatergic excitatory interneurons, involved in pain and touch transmission, include neurons expressing somatostatin (SOM+), cholecystokinin (CCK+), calretinin (CR+), protein kinase C gamma (PKCγ+), and vesicular glutamate transporter 3 (vGlut3+). GABAergic and glycinergic inhibitory interneurons include neurons expressing parvalbumin (PV+), dynorphin (DYN+), galanin (partially overlapping with DYN+), neuronal nitric oxide synthase (nNOS+), and early expressed tyrosin kinase receptor RET (early RET+, partially overlapping with PV+). Nociceptive projection neurons are mostly located in lamina I and relay sensory information to the brain stem and the thalamus. A subpopulation of these neurons expresses the neurokinin receptor NK1.

Primary afferent terminals release primarily glutamate onto dorsal horn neurons. A subclass of Aδ and C fibers also contains peptides, such as Substance P or CGRP, and/or neurotrophic factors (BDNF, GDNF).

The “first synapse” in dorsal horn represents the first opportunity to modify sensory information entering the spinal cord and it is subjected to a very complex and fine modulation. Numerous ion channels and ligand-gated receptors are involved in the modulation of glutamate and peptide release from PAFs, thereby controlling the impact of sensory input on second order neurons. In this review, we will present some recent studies about GABA-mediated presynaptic modulation at the first synapse in spinal cord dorsal horn, focusing on the neural circuits involved.

## 2. GABAergic Inhibition on Primary Afferent Fibers

GABAergic interneurons play a critical role in regulating nociceptive signal strength and separating nociception from touch. Intrathecal application of bicuculline and strychnine (antagonists of GABA_A_ and glycine receptors, respectively) increases responses evoked by exposure to noxious stimuli [5]. According to the gate theory of pain, proposed by Melzack and Wall [6], stimulation of Aβ fibers activate inhibitory interneurons in the dorsal horn, “closing the gate” to nociceptive transmission. Conditions of disinhibition (either pharmacological or induced by persistent pain) “open the gate”, increasing pain response and causing allodynia (i.e., the perception of an innocuous stimulus as noxious).

Presynaptic GABA receptors located on PAF terminals are involved in gating both tactile and noxious stimuli in the dorsal horn. Indeed, GABA receptors of the A and B type are expressed on both nociceptive and non-nociceptive PAFs, where axo-axonic synapses have been described.

GABA_A_ receptors (GABA_A_Rs) are ligand-gated heteropentameric ion channels, most commonly formed by 2α, 2β, and 1γ subunit. The composition of GABA_A_Rs on PAFs is heterogeneous: C fibers express the α2, α3, and α5 subunits, while α1, α2, α3, and α5 are present on myelinated A fiber terminals [7,8,9]. The subunit β3 has been shown as the dominant β subunit expressed in dorsal root ganglion (DRG) neurons of both A and C type: In a mouse line where the β3 subunit is selectively knocked out in primary nociceptors, the GABA current in DRG neurons is decreased and the animals exhibit hypersensitivity to noxious heat and mechanical stimulation [10].

### 2.1. Primary Afferent Depolarization

Presynaptic GABA_A_Rs expressed on PAF terminals mediate primary afferent depolarization (PAD) in spinal cord dorsal horn. This phenomenon, firstly described in muscle afferents [11], consists of a slow dorsal root depolarization, evoked by the stimulation of an adjacent root. Stimulation of PAFs evokes glutamate release that activates GABAergic interneurons. These neurons, in turn, release GABA binding to GABA_A_Rs expressed on PAF terminals.

The cellular mechanisms of PAD have been extensively investigated. Primary sensory neurons exhibit a higher intracellular concentration of chloride than most central neurons. This is caused by the high expression of the transporter NKCC1 (transporting Cl^−^, Na^+^ and K^+^ into the cell) and the low, or even undetectable, expression of KCC2 (expelling Cl^−^ and K^+^ out of the cell) [12,13,14,15,16]. Due to the high Cl^−^ intracellular concentration, the chloride equilibrium potential (E_Cl_) in DRG neurons is about −30 mV [13,17]. Thus, the activation of GABA_A_Rs on PAF terminals produces an outward anion flux, leading to membrane depolarization and generation of PAD.

In physiological conditions, PAD exerts an inhibitory effect on glutamate release, through several possible mechanisms. PAF depolarization can lead to the inactivation of voltage-dependent Na^+^ and Ca^+^ channels, impairing the propagation of action potentials along PAFs and decreasing the calcium influx into the terminals [18,19,20,21,22]. The opening of GABA_A_ channels could also exert a shunting effect on action potential propagation by decreasing membrane resistivity, as shown by experimental evidence and mathematical simulations [23,24,25,26]. Suprathreshold PAF depolarizations can sometimes trigger action potentials that are conducted antidromically, generating dorsal root reflexes [27,28].

Earlier studies have demonstrated the involvement of GABA_A_Rs in the generation of PAD: The GABA_A_ antagonist picrotoxin blocks presynaptic inhibition on spinal monosynaptic reflexes, while iontophoretic application of GABA generates depolarization of group I afferent fibers [29,30]. More recently, Witschi et al. have shown that mice lacking the GABA_A_ α2 subunit specifically in primary nociceptors exhibit a lack of effect of the GABA_A_ modulator diazepam in potentiating PAD and decreasing inflammatory hyperalgesia, confirming the involvement of presynaptic GABA_A_Rs in PAD generation and pain inhibition [31]. Interestingly, neither glycine receptors nor gephyrin clusters have been detected on C fibers expressing GABA_A_Rs, in contrast with inhibitory synapses on postsynaptic neurons [8]. The presence of unclustered GABA_A_Rs on presynaptic terminals suggests a more diffuse mode of inhibition at these synapses, consistent with the slow kinetics of GABA-mediated PAD. Beside GABA_A_Rs, also glutamatergic receptors of the AMPA and NMDA type, expressed on PAF central terminals, have been reported to contribute to the generation of PAD in the spinal cord [32].

PAD has been proposed as one of the most powerful mechanisms of sensory control, producing several effects: (1) reduction in the effectiveness of one sensory input over the others and selective control of convergent inputs; (2) generation of surround inhibition, producing localized reactions to sensory stimuli (a small stimulus to the skin produces PAD in the same spinal cord segment, but also in many rostral and caudal segments, both ipsi- and contra-lateral); and (3) increase of the temporal contrast of a somatic sensory input. Accordingly, PAD mediated by GABA_A_Rs is composed of a phasic and a tonic component (likely mediated by receptors expressing the α5 subunit [33]): The first increases the perception of a sudden stimulus, while the latter represents the ongoing inhibition of slow changes of sensory inputs.

**Figure 2:**
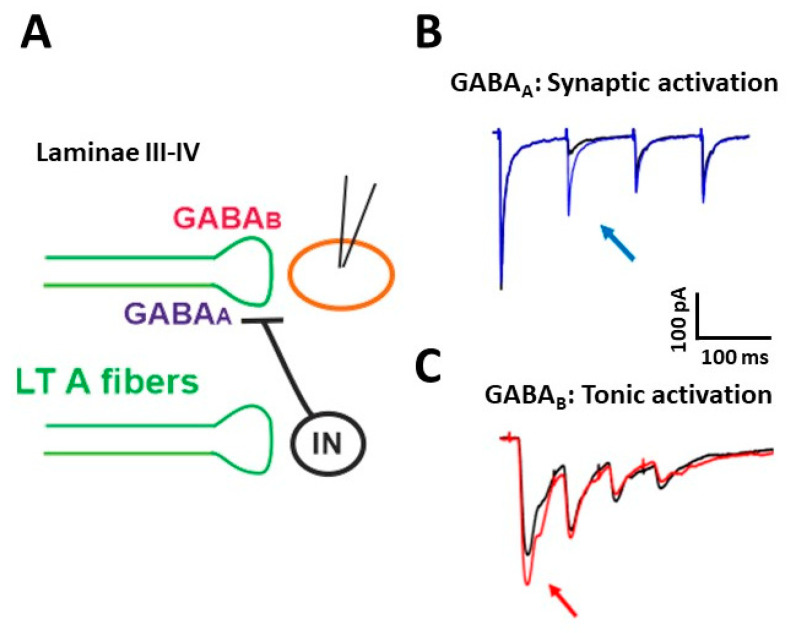
Presynaptic modulation mediated by GABA_A_ and GABA_B_ receptors on low threshold (LT) A fibers in deep dorsal horn (laminae III–IV). (**A**) Schematic representation of the circuit activating presynaptic GABA receptors. GABA_A_ receptors can be recruited by a synaptic mechanism: an inhibitory interneuron (IN) is activated by LT A fibers and releases GABA onto fibers of the same type, causing the inhibition of glutamate release and synaptic depression. GABA_B_Rs (GABA_B_ receptors) can tonically inhibit the release of glutamate from LT fibers. (**B**) Representative traces of EPSCs recorded from a lamina III–IV neuron, evoked by stimulating LT fibers with four pulses at 10 Hz. A strong depression of the second response was evident in control (black trace). Application of the GABA_A_R antagonist gabazine (10 μM) increased the second EPSC (blue trace, arrow) in 10 out of 17 recorded neurons. (**C**) Representative traces of EPSCs, evoked by stimulating LT A fibers with four pulses at 20 Hz. In the presence of the GABA_B_ antagonist CGP 55,845 (5 μM), the first EPSC increased in five out of 13 lamina III–IV neurons (red trace, arrow). Modified with permission from [41].

The functional properties of PAD in muscle afferents and LT cutaneous PAFs have been investigated by several studies [20,27,34,35]. We recently demonstrated that presynaptic GABA_A_Rs are involved in short term synaptic depression during repetitive stimulation of Aβ fibers [36] (Figure 2). We performed electrophysiological experiments on rat spinal cord slices, recording from unidentified laminae III–IV neurons, in voltage-clamp at −70 mV. The dorsal root attached to the slice was electrically stimulated with four pulses at the frequency of 10–20 Hz and intensity of 10–25 μA, recruiting LT A fibers, mainly of the Aβ type. The evoked excitatory postsynaptic currents (EPSCs) showed a strong depression after the first response, which was particularly evident in the second EPSC (Figure 2B, black trace). Application of the GABA_A_ antagonist gabazine unmasked an additional component in the second EPSC (blue trace): This indicates that GABA, released after the first pulse, acts on presynaptic GABA_A_Rs, reducing glutamate release from Aβ fibers at the second stimulus. By mainly affecting the second response in a train of stimuli, presynaptic GABA_A_Rs inhibit glutamate release from PAFs with a high temporal precision, controlling the earliest part of an afferent response to touch

Using a cesium-fluoride intracellular solution, able to block GABA_A_Rs expressed on the recorded neuron, the effect of gabazine on the second EPSC was not abolished, confirming the involvement of presynaptic GABA_A_Rs. Application of strychnine was ineffective in increasing the second EPSC, indicating that glycine receptors are not importantly involved in presynaptic modulation on PAF terminals. By recording at −10 mV from laminae III–IV neurons and stimulating at Aβ fiber threshold, we observed inhibitory postsynaptic currents, mediated by both GABA_A_ and glycine receptors. Thus, GABA_A_Rs modulate the first synapse between Aβ fibers and dorsal horn neurons in two ways: through a negative feedback mechanism at PAF terminals and by a feed-forward control on postsynaptic neurons.

Differently from LT afferent fibers, functional studies about PAD on HT nociceptive fibers are still limited. By using an ex vivo spinal cord preparation, Fernandes et al. [37] recently reported that noxious C-fiber input to rat lamina I neurons (both projection and local neurons) is presynaptically modulated by Aβ, Aδ, and C fibers. Thus, presynaptic inhibition mediated by these different groups of afferents may control the inflow of nociceptive input to superficial dorsal horn, playing a role in nociceptive discrimination and lateral inhibition.

### 2.2. GABA_B_ Receptors as Presynaptic Modulators

In addition to GABA_A_, activation of GABA_B_ receptors (GABA_B_Rs), expressed on nociceptive and non-nociceptive PAF terminals, also contributes to presynaptic inhibition, exerting analgesic and anti-hyperalgesic effects [38]. GABA_B_Rs are G protein-coupled receptors, expressed as obligate heterodimers of the two subunits GABA_B1_ and GABA_B2_. The GABA_B1_ isoforms 1a and 1b, together with the subunit GABA_B2_, have been found in small and large DRG neurons and in the spinal cord, both at PAF terminals and on dorsal horn neurons [39,40,41]. Endogenous or exogenous activation of GABA_B_Rs in superficial dorsal horn causes both pre- and postsynaptic effects. Electrophysiological studies performed on rats have shown that presynaptic GABA_B_Rs inhibit pinch- and touch-evoked synaptic responses in vivo [42] and decrease glutamate and peptide release from A- and C-type PAFs and dorsal horn neurons [40,43,44,45,46]. The inhibitory effect of GABA_B_Rs on transmitter release is due to the concurrent inhibition of presynaptic calcium channels [47,48] and release machinery downstream of calcium entry into the nerve terminals [49].

By performing electrophysiological experiments similar to those described above, we showed that the block of GABA_B_Rs increases the first EPSC in a train of four stimuli, recorded from lamina III–IV neurons [41] (Figure 2C, red trace). This suggests that, differently from GABA_A_Rs, which require the release of GABA through synaptic activation, GABA_B_Rs are tonically activated, confirming the finding of a previous study performed in lamina II [50].

## 3. Dorsal Horn Circuits Involved in GABAergic Presynaptic Inhibition

The majority of inputs to PAF central terminals are represented by local neurons. Axo-axonic synapses formed by GABAergic interneurons onto PAF terminals have been detected on both muscle and cutaneous PAFs [2,20]. Some of these synapses are localized in complex synaptic structures named glomeruli [51], containing central projections of PAFs, GABAergic axo-axonic inputs, and likely also dendro-axonic synaptic inputs. PAD is believed to be originated at GABAergic axo-axonic synapses onto PAFs, but also at extrasynaptic sites, where GABA could diffuse by volume transmission from synapses or en passant varicosities located near PAF terminals [52].

### 3.1. Presynaptic Inhibition of Tactile Stimuli

**Figure 3:**
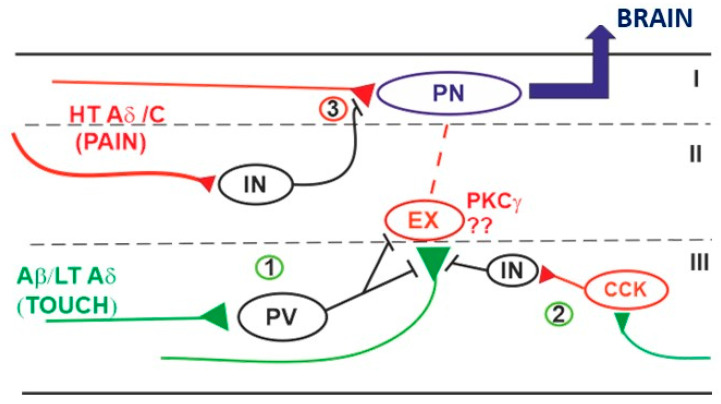
Schematic representation of neural circuits involved in GABAergic presynaptic inhibition in dorsal horn. (**1**) Parvalbumin-positive neurons (PV+) receive low threshold tactile inputs and inhibit both A fiber terminals and some populations of excitatory interneurons (EX) (such as protein kinase C gamma neurons, PKCγ+), connected (directly or indirectly) to nociceptive projection neurons (PN). Loss of activity of PV+ determines the activation of the excitatory interneurons by tactile stimuli and their transmission to nociceptive PNs, generating mechanical allodynia [54,55]. (**2**) A disynaptic circuit could also be involved in A fiber inhibition, by recruiting an excitatory interneuron (possibly cholecystokinin neuron, CCK+), and an unknown inhibitory interneuron [56]. 3. HT (high threshold) nociceptive fibers are subjected to presynaptic inhibition by GABAergic interneurons. The nature of these neurons is still not identified, although early RET+ neurons (expressing the tyrosin kinase receptor RET in the early postnatal period) could be involved [57].

The nature of some of the GABAergic interneurons mediating PAD has been highlighted by recent studies. In particular, parvalbumin (PV+) neurons located in mouse lamina II inner or lamina III form numerous axo-axonic synapses onto VGlut1 (Vesicular glutamate transporter 1) expressing myelinated PAFs (including LT mechanoreceptors and non-nociceptive Aδ down-hair afferents), both at glomerular and non-glomerular complexes [53]. As shown by Boyle et al. [54], opto-stimulation of PV+ neurons prior to dorsal root electrical stimulation causes a time-dependent reduction of the amplitude of evoked EPSCs recorded from dorsal horn neurons, confirming the involvement of PV+ neurons in presynaptic inhibition. PV+ neurons receive monosynaptic inputs from LT myelinated fibers and form inhibitory synapses also with dorsal horn excitatory interneurons, such as PKCγ+, that convey nociceptive information to projection neurons [55]. So, according to the gate theory of pain, PV+ neurons provide in normal conditions a powerful inhibition of excitatory interneurons, preventing innocuous mechanical input from activating nociceptive mechanical circuits [54,55] (Figure 3).

### 3.2. Presynaptic Modulation of Nociceptive Fibers

The nature of GABAergic interneurons mediating PAD on nociceptive fibers is still largely unknown. Cui et al. [57] have characterized a population of dorsal horn inhibitory neurons located in mouse deep dorsal horn (laminae III–IV), expressing neonatally the tyrosine kinase receptor RET (early RET+). These neurons may receive inputs from both A and C fibers and inhibit dorsal horn excitatory interneurons, such as PKCγ+ and SST+ (somatostatin expressing neurons). Early RET+ neurons make presynaptic inhibitory synapses onto PAF terminals; their optogenetic activation inhibits Aβ or C fiber-mediated responses in dorsal horn neurons, through the activation of presynaptic GABA_B_ receptors. This suggests that early RET+ neurons are involved in presynaptic inhibition of both pain and touch, likely mediating the cross talk between the two sensory modalities.

An interesting and complex mechanism of presynaptic inhibition involving both GABA_A_ and NMDA receptors has been recently described by Zimmerman et al. [56]. In the mouse ex vivo hemisected spinal cord preparation, optogenetic activation of distinct groups of fibers produces three mechanisms of presynaptic inhibition: two homotypic, GABA_A_-dependent modulations, where activation of LT tactile or HT nociceptive fibers evokes PAD in fibers of the same type; one heterotypic, NMDA-dependent mechanism, evoked by nociceptive stimulation and inhibiting tactile fibers. GABA- and NMDA-dependent presynaptic inhibitions seem to be functionally distinct: They are exerted in different skin regions and only the GABA_A_-dependent modulation is required for texture discrimination.

## 4. GABA Release from Descending Fibers and Astrocytes

Although GABAergic interneurons seem to be the principal source of GABA for presynaptic inhibition on PAFs, other possible sources of GABA have been described.

PAF terminals receive inputs from fibers descending from brain stem and hypothalamus, releasing different amines, such as serotonin, noradrenaline, and dopamine, involved in presynaptic modulation [58,59,60,61]. A large proportion of fibers descending from the brain stem rostral ventral medulla (RVM) are represented by descending GABAergic fibers, most of which release also enkephalin. Opto-activation of dual GABAergic/enkephalinergic RVM neurons evokes dorsal root potentials with short latencies in mouse cervical dorsal roots, suggesting that these descending fibers provide functional and direct presynaptic inputs to PAFs [62]. Ablation of GABAergic/enkephalinergic RVM neurons, by using a Cre-dependent active caspase system, causes hypersensitivity to both heat and mechanical stimuli, whereas chemogenetic activation of these neurons produces antinociception. François et al. [63] characterized a group of GABAergic fibers originating in RVM, which do not express enkephalin and form inhibitory synapses onto enkephalinergic interneurons in the dorsal horn. Opto-stimulation of this descending pathway causes facilitation of mechanical pain and could be involved in the mechanical hypersensitivity produced by chronic stress.

A descending cortico-spinal system involved in sensory modulation has also been described in rats [64]. This pathway, originating in the sensorimotor cortex, inhibits C-fiber nociceptive responses through the activation of presynaptic GABA_A_Rs. Cortical modulation of spinal cord sensory processing could be particularly important for motor control: At the onset of voluntary movement this system would be involved in the selection of sensory information, required for the correct execution of the motor task.

Dorsal horn astrocytes respond to nociceptive fibers’ stimulation with calcium oscillations [65] and can release several gliotransmitters, including glutamate and purines [66,67]. Recently, dorsal horn astrocytes in turtle spinal cord have been reported to release GABA in response to glutamate application [68]. Interestingly, the time course of GABA release from astrocytes is similar to the slow component of PAD generated by a non-spiking circuit [69], suggesting that astrocytes could be involved in presynaptic inhibition of PAFs in normal and/or pathological conditions.

## 5. Modifications of Presynaptic Inhibition in Chronic Pain

Persistent pain can be generated by a tissue or nerve injury, leading to inflammatory or neuropathic pain, respectively. In both conditions sensitization to pain occurs, causing hyperalgesia (the intensity of a noxious stimulus is increased) and allodynia (an innocuous stimulus, mechanical or thermal, is perceived as painful).

A loss of inhibition mediated by GABA has been observed in both inflammatory and neuropathic pain: As evidenced in several animal models of chronic pain, both pre- and postsynaptic inhibitions become less efficient, leading the dorsal horn to a state of hyperexcitability [4,70,71].

Several studies have shown that PAD is subjected to complex modifications in chronic pain conditions. In inflammatory pain, a switch from presynaptic inhibition to excitation has been reported: The amplitude of PAD is increased in both nociceptive and non-nociceptive fibers after tissue injury, and this could be sufficient to generate action potential firing at PAF terminals and dorsal root reflexes [27,72]. Orthodromically propagated action potentials may enhance glutamate and peptide release from central nociceptive terminals, causing excitation of spinal second order neurons [72,73]. Action potentials propagated antidromically to the periphery have been involved in neurogenic inflammation in experimental arthritis or after intradermal injection of capsaicin [74,75,76]. Changes of chloride homeostasis and GABA_A_R activity in DRG neurons could be involved in the increase of PAD amplitude observed after inflammation. Accordingly, inflammatory mediators cause the increase of NKCC1 activity and intracellular chloride concentration in DRG neurons in vitro [77], painful stimulation enhances phosphorylation and membrane mobilization of NKCC1 [78], and GABA sensitivity of DRG neurons is increased in a model of inflammatory joint pain [79].

Following nerve injury, PAD amplitude is generally reduced and presynaptic inhibition is diminished [70,71]. Several factors seem to contribute to PAD modifications in neuropathic pain: (1) decrease of GABA synthesis, due to the reduced expression of the GABA synthetizing enzyme GAD65 in dorsal horn inhibitory interneurons [8,80]; (2) increase of NKCC1 expression and activity, causing a depolarizing shift of E_Cl_ [22,81,82]; and (3) reduction of GABA_A_ conductance and downregulation of γ2, α2, and α1 subunits on DRG neurons, observed after nerve ligation, chronic constriction, and crush nerve injury [10,83,84,85,86]. Consistently, the selective downregulation of the α2 subunit in DRGs worsens thermal and mechanical hypersensitivity in crush-injured animals and induces pain hypersensitivity in sham animals, while upregulation of endogenous GABA alleviates neuropathic pain [86].

A decreased activity of inhibitory circuits in dorsal horn has been correlated to the development of mechanical allodynia in both inflammatory and neuropathic pain. Previous studies have identified a dorsally directed excitatory polysynaptic pathway conveying tactile input from LT PAFs to lamina I projection neurons [87,88]. In physiological conditions, this pathway is inhibited by interneurons releasing GABA and glycine. In inflammatory and neuropathic pain models, the efficacy of spinal inhibition is reduced and the tactile input is allowed to reach the nociceptive projection neurons, causing allodynia. Duan et al. [89] showed that inhibitory DYN+ neurons, receiving Aβ-mediated synaptic inputs, play an important role in gating the tactile input to the excitatory SOM+ neurons in lamina II. Selective ablation of DYN+ neurons (by using an intersectional genetic strategy leading to the neuron-specific expression of the diphtheria toxin receptor) generates spontaneous mechanical allodynia and Aβ fiber-mediated action potential firing in superficial dorsal horn neurons. A more recent study [90] identified specific subpopulations of excitatory interneurons involved in different forms of mechanical allodynia: By using chemogenetic approaches, allowing the selective inhibition of different classes of neurons, the authors demonstrated that CR+, CCK+, and V-Glut3+ neurons are involved in mechanical allodynia in inflammatory models, while PKCγ+ (together with CCK+ and V-Glut3+) neurons are critical for allodynia induced by nerve injury.

Alterations of presynaptic inhibition on LT A fibers could play an important role in the genesis of mechanical allodynia. A reduction of GABA-mediated inhibition on these fibers would cause the increase of glutamate release onto excitatory interneurons, activating a relay circuit that conveys the tactile input to nociceptive projecting neurons (Figure 3). Inhibitory PV+ neurons, forming axo-axonic synapses with LT A fibers, seem to be importantly involved in this mechanism: Nerve injury reduces the excitability of PV+ neurons, their chemogenetic activation significantly attenuates allodynia induced by nerve injury, and saporin-mediated ablation of PV+ neurons induces a form of mechanical allodynia that is PKCγ-dependent [55].

Although initially a neurodegenerative process had been proposed for the loss of GABAergic inhibition in the dorsal horn in chronic pain, recent studies have shown that nerve injury does not cause loss of PV+ neurons or changes in their synaptic connectivity [54]. However, peripheral axotomy determines a reduction of excitability in PV+ neurons, and larger currents become necessary to elicit the sustained tonic action potential discharge typical of these neurons. A decrease of the expression of PV, a calcium-buffering protein, could contribute to this effect: The rise of intracellular calcium, subsequent to PV decrease, could activate calcium-dependent potassium channels and prevent the fast spiking activity in this neuron population [55]. A reduction in the excitatory drive from Aβ fibers onto inhibitory neurons could also be responsible for the loss of presynaptic inhibition observed in neuropathic pain models. Accordingly, a decrease of evoked and miniature excitatory postsynaptic currents was observed in inhibitory interneurons after injury [91].

Changes of presynaptic inhibition on nociceptive fibers have been studied in less detail. Specific ablation of early RET+ neurons, through injection of a Cre-dependent virus expressing diphtheria toxin, increases basal nociception and leads to more severe inflammatory and neuropathic pain, whereas opto-stimulation of these neurons produces antinociception [57]. Further studies are required to determine whether modifications in the activity of these or other inhibitory neurons affects presynaptic inhibition on HT nociceptive fibers.

## 6. Conclusions and Future Perspectives

During the last decade substantial progress has been made in the characterization of spinal cord dorsal horn circuits involved in sensory transmission. By combining advanced techniques, such as genetic labeling, opto- and chemogenetic manipulation, and specific neuronal ablation, several neuron populations have been characterized and their role in sensory perception has been established. As described here, some of these neurons are involved in presynaptic modulation of PAFs and changes in their functional properties could contribute to the hyperalgesic and allodynic states related to chronic pain.

Despite these advances, many aspects need further investigation. Neuronal circuits mediating presynaptic inhibition on PAFs are largely unknown, especially those modulating nociceptive fibers. More information is also needed about the modifications occurring to these circuits after tissue or nerve injury. Furthermore, the interplay between different presynaptic receptors on PAF central terminals is still not clear. Although many receptors have been identified, both ionotropic and metabotropic, their role in acute nociception and in states of pain sensitization is still not understood. NMDA receptors, for example, are expressed on PAF terminals and could mediate PAD, but it is still not clear if they are functional in normal conditions or become active only in pathological conditions [92].

The characterization of functional and pharmacological properties of presynaptic receptors will be also important to develop more specific analgesic treatments. Recently, a partial agonist at the benzodiazepine binding sites of GABA_A_Rs expressing the subunits α2, α3, or α5 (particularly relevant for spinal analgesia) was tested in preclinical assays and human studies [93,94]. Lacking activity at α1 GABA_A_Rs, this compound could exert analgesic effects without the unwanted effects of classic benzodiazepines, such as sedation, memory impairment, tolerance, and addiction.

GABA_B_Rs are also importantly involved in presynaptic inhibition in superficial dorsal horn, exerting antinociceptive effects. The GABA_B_ agonist baclofen is primarily used for the treatment of severe spasticity, while its use as an analgesic drug is prevented by the numerous side effects [95]. The development of positive allosteric modulators (PAMs), which enhance the activity of GABA_B_Rs in the presence of endogenous or exogenous orthosteric agonists, could represent a promising strategy [38]. While recently developed PAMs (such as ADX71441) seem to exert significant antinociceptive activity in inflammatory pain models by potentiating the endogenous activation of GABA_B_Rs [96], in neuropathic animals the allosteric modulator rac-BHFF has no efficacy in increasing the endogenous GABA_B_ tone, but it might be useful to enhance baclofen-mediated analgesia at low baclofen doses [97].

Finally, the role of glia, and especially of astrocytes, in dorsal horn sensory processing is still poorly understood. As evidenced in several brain areas, gliotransmitters released by astrocytes play a critical role in modulating neuronal activity, in both physiological and pathological conditions. Future studies combining electrophysiology, calcium imaging, and selective activation of dorsal horn astrocytes will be important to establish their role in pre- and postsynaptic modulation of neuronal activity.
